# Disability perceived by primary care patients with posterior canal benign paroxysmal positional vertigo

**DOI:** 10.1186/s12875-019-1035-3

**Published:** 2019-11-13

**Authors:** Ricard Carrillo Muñoz, José Luis Ballve Moreno, Iván Villar Balboa, Yolanda Rando Matos, Oriol Cunillera Puertolas, Jesús Almeda Ortega, Estrella Rodero Perez, Xavier Monteverde Curto, Carles Rubio Ripollès, Noemí Moreno Farres, Austria Matos Mendez, Jean Carlos Gomez Nova, Marta Bardina Santos, Johan Josué Villarreal Miñano, Diana Lizzeth Pacheco Erazo, Anabella María Hernández Sánchez, Grupo de estudio del vértigo en atención primaria Florida, José Luis Ballve Moreno, Yolanda Rando Matos, Estrella Rodero Pérez, Xavier Monteverde Curto, Carles Rubio Ripollès, Noemí Moreno Farres, Jean Carlos Gómez Nova, Johan Josué Villarreal Miñano, Diana Lizzeth Pacheco Erazo, Raquel Adroer Martori, Anna Aguilar Margalejo, Olga Lucia Arias Agudelo, Silvia Cañadas Crespo, Laura Illamola Martín, Marta Sarró Maluquer, Lluís Solsona Díaz, Rosa Sorando Alastruey, Ricard Carrillo Muñoz, Iván Villar Balboa, Austria Matos Méndez, Marta Bardina Santos, Oriol Cunillera Puertolas, Jesús Almeda Ortega

**Affiliations:** 1 Equip d’Atenció Primària Florida Sud, Institut Català de la Salut, Hospitalet de Llobregat Barcelona, Spain; 2 Equip d’Atenció Primària Florida Nord, Institut Català de la Salut, Hospitalet de Llobregat Barcelona, Spain; 3 Universitat Autònoma de Barcelona, Bellaterra, Cerdanyola del Vallès Barcelona, Spain; 4 Unitat de Suport a la Recerca Costa de Ponent, Institut Universitari d’Investigació en Atenció Primària Jordi Gol (IDIAPJGol), Cornellà Barcelona, Spain; 5 Hospital General de Hospitalet, Hospitalet de Llobregat Barcelona, Spain

**Keywords:** Benign paroxysmal positional vertigo, Primary care, Quality of life

## Abstract

**Background:**

Benign paroxysmal positional vertigo (BPPV) is the most common cause of vertigo. Little is known on how posterior canal BPPV affects health-related quality of life in patients diagnosed and treated at primary care facilities or on whether patients with subjective and objective disease perceive the effects differently. This study was designed to describe how patients diagnosed with posterior canal BPPV in primary care perceive disability.

**Methods:**

Cross-sectional descriptive study performed at two urban primary care centers. Participants were patients aged 18 years or older with suspected posterior canal BPPV recruited for baseline evaluation in a clinical trial on the effectiveness of the Epley maneuver in primary care. The recruitment period was from November 2012 to January 2015. Perceived disability was evaluated using the Dizziness Handicap Inventory – Screening version (DHI-S). Other variables collected were age and sex, a history or diagnosis of anxiety or depression, treatment with antidepressants and/or anxiolytics, and results of the Dix-Hallpike (DH) test, which was considered positive when it triggered vertigo with or without nystagmus and negative when it triggered neither.

**Results:**

The DH test was positive in 134 patients, 40.30% of whom had objective BPPV (vertigo with nystagmus). The median age of the patients was 52 years (interquartile range [IQR], 39.00–68.50 years) and 76.1% were women. The median total score on the DHI-S was 16 out of 40 (IQR, 8.00–22.00). Scores were higher (greater perceived disability) in women (*p* < 0.001) and patients with subjective BPPV (vertigo without nystagmus) (*p* = 0.033). The items perceived as causing the greatest disability were feeling depressed (67.1%) and worsening of the condition on turning over in bed (88%).

**Conclusions:**

Patients diagnosed with posterior canal BPPV in primary care perceive their condition as a disability according to DHI-S scores, with higher levels of disability reported by women and patients with subjective BPPV. Feelings of depression and turning over in bed were associated with the greatest perceived difficulties.

**Trial registration:**

ClinicalTrials.gov Identifier: NCT01969513. Retrospectively registered. First Posted: October 25, 2013. https://clinicaltrials.gov/ct2/show/NCT01969513

## Background

Benign paroxysmal positional vertigo (BPPV) is the most common cause of vertigo. It has an annual incidence of between 10.7 and 140 cases per 100,000 inhabitants [1] and a lifetime prevalence of 2.4% [2]. Between 60 and 90% of cases involve the posterior canal [3]. Posterior canal BPPV can be diagnosed in primary care with a targeted history, a basic physical examination, [4]. This test is considered positive when it triggers both vertigo and nystagmus (objective BPPV), although some authors consider that a diagnosis can also be made when it triggers vertigo only (subjective BPPV) [5]. Subjective BPPV accounts for approximately 11.5 to 48% of all cases of BPPV [6]. Studies comparing patients with subjective and objective BPPV have not found differences in terms of demographics or clinical characteristics [7] but little is known on how the two entities affect the quality of life of patients seen in primary care practices.

Patients with BPPV often have physical, functional, and even emotional disabilities that can affect their family or social lives [8]. They are at an increased risk of falls [9], particularly if they are elderly [10], and may also experience psychological symptoms that can affect their daily activities [11]. The resulting distress can have a negative impact on health-related quality of life (HRQOL).

Being exposed to emotional stress increases the odds of getting an episode of Menière’s disease during the following hours in diagnosed patients [12, 13], and anxiety it is also a risk factor for poor prognosis in primary care patients presenting with dizziness [13]*.* Dizziness can also be brought about by stressful events in the previous year [14], and patients with BPPV have been found to score higher in depression evaluation tests [15].

The impact of BPPV on HRQOL can be assessed using a variety of standardized questionnaires. The most widely used questionnaire is the Dizziness Handicap inventory (DHI) [16]. The original version has 25 items with three possible answers (yes, sometimes, and no) but two shorter versions have been designed: the DHI-SF (short form), which has 13 items each with two possible answers [17], and the DHI-S (screening version), which has 10 items with the same three answers as the original questionnaire [18]. The DHI-S is strongly correlated with the original DHI (r = 0.86) and has high internal consistency (test-retest r = 0.95). It is a self-assessment questionnaire that can be completed in about 4 to 5 min and is therefore suitable for use in settings with large volumes of patients, such as primary care. It has also been validated for use in Spanish [19]. A review comparing the DHI, the DHI-SF, and the DHI-S concluded that the DHI-S was the best scale to use because of its shorter length and close correlation with the original questionnaire [20]. The authors also discouraged the use of domain scores in favor of total score.

Little is known, however, on how BPPV affects HRQOL in patients diagnosed and treated at primary care facilities or on whether patients with subjective and objective disease perceive the effects differently. Moreover, very few studies have used the DHI-S to evaluate self-perceived disability in patients with BPPV, even though it is one of the simplest psychometric tests available [19]. As indicated in the recently updated Clinical Practice Guideline on BPPV, research on the impact of BPPV on HRQOL must continue [1].

The aim of this study was to describe self-perceived disability using the DHI-S in patients with BPPV diagnosed in primary care prior to treatment with the Epley maneuver.

## Methods

We performed a cross-sectional descriptive study of two primary care centers with 26 general practitioners (GPs) serving an approximate population of 38,305 people in L’Hospitalet de Llobregat (Barcelona, Spain). The GPs received 2 h of training from an otolaryngologist in the adequate management of patients with vertigo and the correct application and interpretation of the DH test.

Patients aged 18 years or older with a suspected diagnosis of BPPV recruited for a baseline visit within a randomized clinical trial designed to demonstrate the effectiveness of the Epley maneuver for treating posterior canal BPPV in primary care were eligible for inclusion. The protocol of the trial has been published elsewhere. [21]

**Fig. 1:**
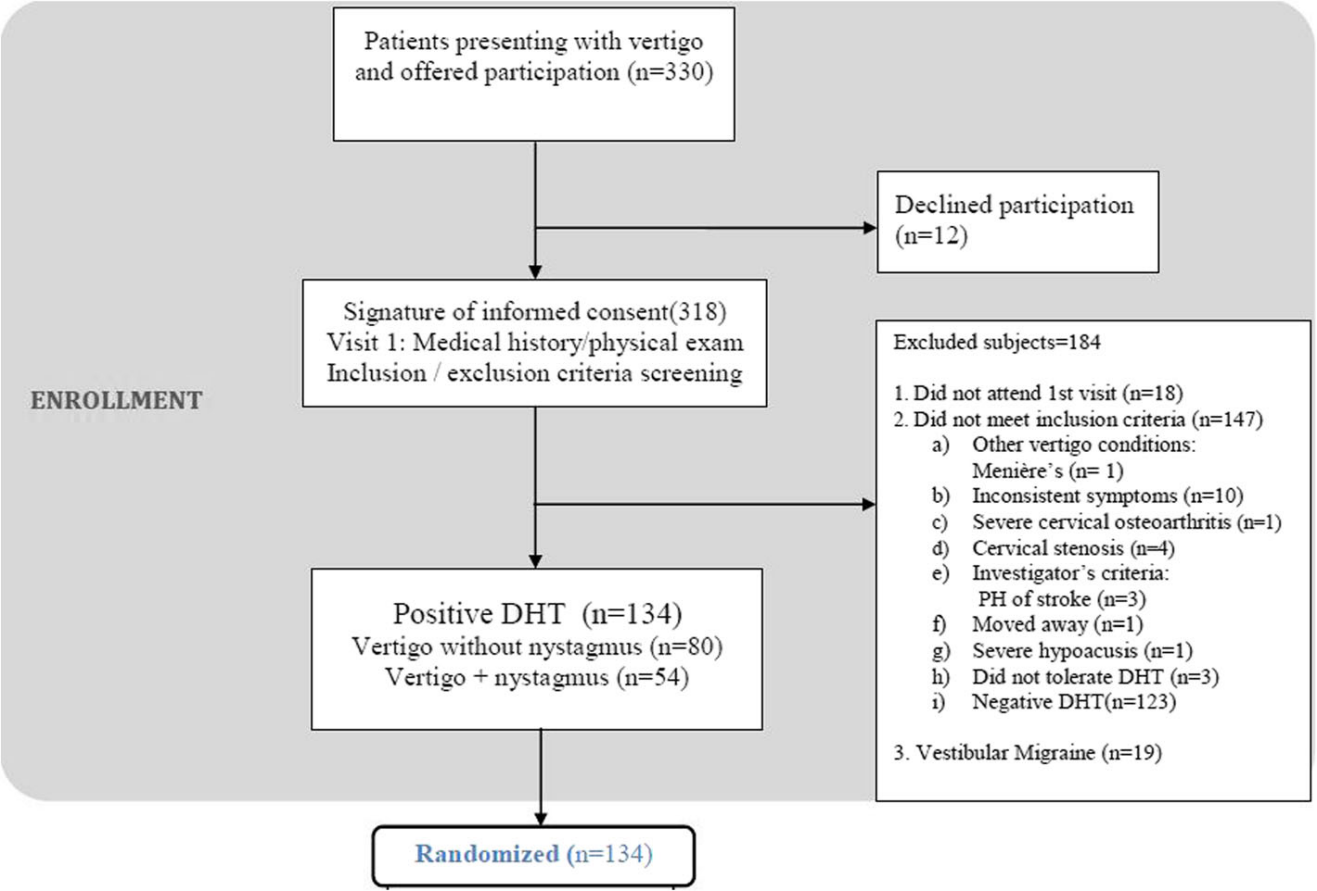
Flowchart of the study. DH = Dix-Hallpike

Patients with a positive DH test were included and those whose results suggested involvement of a portion of the semicircular canal other than the posterior canal or vertigo of central origin were excluded and referred to an otolaryngologist. The rest of the inclusion and exclusion criteria can be consulted in the trial protocol [21] and flow chart (Fig. 1). Nineteen of the patients initially recruited were subsequently excluded because they met the criteria for probable vestibular migraine. This analysis was performed retrospectively in light of increasing evidence on the high prevalence of vestibular migraine and its overlapping symptoms with BPPV [22].

Patients were consecutively recruited by the participating GPs and referred for baseline evaluation within a maximum of 10 days by one of six GPs in the clinical trial team*.* All patients were being treated with betahistine 8 mg 8- hourly at the initial visit, along with the instruction of P.R.N. use (up to 3 times a day) until improvement of symptoms*.* The recruitment period was between November 2012 and January 2015.

All the patients had their history taken and underwent a full physical examination and electronic chart review. Disability was assessed using the total DHI-S score as the dependent (outcome) variable. The DHI-S has 10 items that are graded with a score of 0 (for the answer “no”), 2 (“sometimes”), or 4 (“yes”). The minimum score thus is 0 (no disability) while the maximum score is 40 (worst possible self-perceived disability). The independent variables were age, age group (< 65 y vs ≥65 y), sex, DH test result (vertigo only vs vertigo with nystagmus); a previous history of anxiety and/or depression; and treatment with antidepressants or anxiolytics.

The distribution of DHI-S responses was studied by describing the number of *yes*, *no*, and *sometimes* answers to the 10 items for the sample as a whole and stratified by age, sex, presence of nystagmus, and diagnosis of anxiety and/or depression. Total scores were described using medians and interquartile range (IQR) for the full sample and for subgroups stratified by age, sex, nystagmus, and anxiety and/or depression (stratified by sex).

The sample was described using median and IQR for age and absolute and relative frequencies for presence of binary variables and for each of the categories in the categorical variables.

Finally, the Fisher exact test was used to compare the distribution of DHI-S responses according to the study variables. The distribution of total scores by subgroup was compared using the Wilcoxon Rank Sum test. Statistical significance was set at a *p* value of 0.05 or less.

## Results

Of the 330 patients with suspected BPPV, 165 were excluded at the baseline visit (mostly because of a negative DH test) and 19 were excluded retrospectively as they met the criteria for probable vestibular migraine (Fig. 1). We therefore studied 134 patients (40.6%). Of these, 54 (40.30%) had objective BPPV (vertigo with nystagmus).

The median age for the sample was 52 years (IQR, 39.00–68.50) and 76.12% were women. The prevalence of anxiety and depression and the use of benzodiazepines and antidepressants are shown in Table 1.

**Table 1:** Characteristics of primary care patients with symptoms of benign paroxysmal positional vertigo and a positive Dix-Hallpike test

	No. of patients	
Age, median (IQR), y	134	52.00 [38.25–68.00]
Age group	134	
*< 65 y*		92 (68.66%)
*≥ 65 y*		42 (31.34%)
Sex	134	
*Male*		32 (23.88%)
*Female*		102 (76.12%)
Nystagmus	134	
*No (S-BPPV)*		80 (59.70%)
*Yes (O-BPPV)*		54 (40.30%)
Comorbidities		
Anxiety	130*	33 (25.38%)
Depression	130*	30 (23.08%)
Drugs		
Benzodiazepines	134	24 (17.91%)
Antidepressants	134	27 (20.15%)
Health-related quality of life		
Total DHI-S score	134	16.00 [8.00–22.00]

*Data missing for 4 patients

Abbreviations: *DHI-S* Dizziness Handicap Inventory Screening version, *IQR* interquartile range, *O-BPPV* objective benign paroxysmal positional vertigo, *S-BPPV* subjective benign paroxysmal positional vertigo

Table 2 summarizes the responses to the DHI-S. The items for which the fewest patients reported difficulties were item 6 (“Because of your problem, do you restrict your travel for business or holidays?”) and item 9 (“Because of your problem, have you ever been embarrassed in front of others”?), with over 70% of respondents saying they did not experience problems in this area. By contrast, the areas that caused the greatest difficulties were items 5 (Does turning over in bed worsen your problem?) and 1 (Because of your problem, do you feel depressed?), with 74.6% of patients answering “yes” or “sometimes” when asked if turning over in bed increased their problem and 67.1% answering “yes” or “sometimes” when asked if their problem made them feel depressed.

**Table 2:** Distribution of responses to the Dizziness Handicap Inventory – Screening version for primary care patients with benign paroxysmal positional vertigo

	No	Sometimes	Yes
1. Because of your problem, do you feel depressed?	44 (32.8%)	35 (26.1%)	55 (41.0%)
2. Does stepping off the sidewalk worsen your problem?	63 (47.0%)	24 (17.9%)	47 (35.1%)
3. Because of your problem, is it difficult to concentrate?	68 (50.7%)	26 (19.4%)	40 (29.9%)
4. Because of your problem, is it difficult for you to walk around the house in the dark?	76 (56.7%)	20 (14.9%)	38 (28.4%)
5. Does turning over in bed worsen your problem?	16 (11.9%)	18 (13.4%)	100 (74.6%)
6. Because of your problem, do you restrict your travel for business or holidays?	95 (70.9%)	21 (15.7%)	18 (13.4%)
7. Does your problem affect your job or household responsibilities?	54 (40.3%)	33 (24.6%)	47 (35.1%)
8. Because of your problem, are you afraid to leave your home without having someone with you?	85 (63.4%)	20 (14.9%)	29 (21.6%)
9. Because of your problem, have you ever been embarrassed in front of others?	96 (71.6%)	26 (19.4%)	12 (9.0%)
10. Because of your problem, have you reduced your social activities such as going out to dinner, going to movies, or dancing at parties?	83 (61.9%)	20 (14.9%)	31 (23.1%)

Although we used the Spanish version of the questionnaire, we have included the original English questionnaire for comprehension purposes. Data shown as number and percentage of respondents

The median DHI-S total score was 16 (IQR, 8.00–22.00) (Table 1). Statistically significant higher scores (greater perceived disability) were detected in women and in patients without nystagmus in the DH test (*p* < 0.001 and *p* = 0.033, respectively). No bivariate association was detected for age or for a diagnosis of anxiety or depression stratified by sex with DHI score (Table 3).

**Table 3:** Total median score on the Dizziness Handicap Inventory – Screening version by subgroups of primary care patients with symptoms of benign paroxysmal positional vertigo

	Median [IQR]	*P* value*
Age group		0.187
*[< 65 y) (n = 92)*	16.0 [10.0–24.0]	
*[≥65 y) (n = 42)*	12.0 [8.0–20.0]	
Sex		**< 0.001**
*Men (n = 32)*	10.0 [6.0–14.0]	
*Women (n = 102)*	16.0 [10.5–24.0]	
Presence of nystagmus		**0.033**
*No (S-BPPV) (n = 80)*	16.0 [10.0–24.0]	
*Yes (O-BPPV) (n = 54)*	12.0 [8.0–18.0]	
History of anxiety and/or depression in men		0.347
*No (n = 207)*	11.0 [7.5–14.0]	
*Yes (n = 6)*	8.0 [6.0–10.0]	
History of anxiety and/or depression in women		0.648
*No (n = 59)*	16.0 [10.0–24.0]	
*Yes (n = 41)*	16.0 [12.0–24.0]	

* Calculated using the Wilcoxon test (statistical significance: p ≤ 0.05)

Abbreviations: *IQR* interquartile range, *O-BPPV* objective benign paroxysmal positional vertigo, *S-BPPV* subjective benign paroxysmal positional vertigo

Statistically significant results are presented in bold

Responses to DHI-S items according to age, sex, objective and subjective BPPV, and a history of anxiety and/or depression are shown in Tables 4 to 7. The distribution of responses was very similar in the two age groups (Table 4). On comparing men and women (Table 5) several significant differences were detected. Women reported significantly greater difficulties in stepping off the sidewalk (*p* = 0.015), concentrating (*p* = 0.029), working and fulfilling their household responsibilities (*p* < 0.001), and leaving their home alone (*p* = 0.024).

**Table 4:** Distribution of responses to the Dizziness Handicap Inventory – Screening version for primary care patients with benign paroxysmal positional vertigo according to age group (< 65 vs ≥ 65 y)

	(< 65 y) (*n* = 92)	[≥65–96 y) (*n* = 42)	*P* value
1. Because of your problem, do you feel depressed?			0.588
No	28 (30.4%)	16 (38.1%)	
Sometimes	26 (28.3%)	9 (21.4%)	
Yes	38 (41.3%)	17 (40.5%)	
2. Does stepping off the sidewalk worsen your problem?			0.909
No	42 (45.7%)	21 (50.0%)	
Sometimes	17 (18.5%)	7 (16.7%)	
Yes	33 (35.9%)	14 (33.3%)	
3. Because of your problem, is it difficult to concentrate?			0.108
No	41 (44.6%)	27 (64.3%)	
Sometimes	19 (20.7%)	7 (16.7%)	
Yes	32 (34.8%)	8 (19.0%)	
4. Because of your problem, is it difficult for you to walk around the house in the dark?			0.719
No	50 (54.3%)	26 (61.9%)	
Sometimes	14 (15.2%)	6 (14.3%)	
Yes	28 (30.4%)	10 (23.8%)	
5. Does turning over in bed worsen your problem?			0.712
No	11 (12.0%)	5 (11.9%)	
Sometimes	14 (15.2%)	4 (9.5%)	
Yes	67 (72.8%)	33 (78.6%)	
6. Because of your problem, do you restrict your travel for business or holidays?			0.863
No	64 (69.6%)	31 (73.8%)	
Sometimes	15 (16.3%)	6 (14.3%)	
Yes	13 (14,1%)	5 (11,9%)	
7. Does your problem affect your job or household responsibilities?			0.733
No	35 (38.0%)	19 (45.2%)	
Sometimes	23 (25.0%)	10 (23.8%)	
Yes	34 (37.0%)	13 (31.0%)	
8. Because of your problem, are you afraid to leave your home without having someone with you?			0.234
No	55 (59.8%)	30 (71.4%)	
Sometimes	17 (18.5%)	3 (7.1%)	
Yes	20 (21.7%)	9 (21.4%)	
9. Because of your problem, have you ever been embarrassed in front of others?			0.332
No	63 (68.5%)	33 (78.6%)	
Sometimes	21 (22.8%)	5 (11.9%)	
Yes	8 (8.7%)	4 (9.5%)	
10. Because of your problem, have you reduced your social activities such as going out to dinner, going to movies, or dancing at parties?			0.545
No	54 (58.7%)	29 (69.0%)	
Sometimes	15 (16.3%)	5 (11.9%)	
Yes	23 (25.0%)	8 (19.0%)	

Although we used the Spanish version of the questionnaire, we have included the original English questionnaire for comprehension purposes. Data shown as number and percentage of respondents

Statistically significant results are presented in bold

**Table 5:** Distribution of responses to the Dizziness Handicap Inventory – Screening version for 134 primary care patients with benign paroxysmal positional vertigo according to sex

	Men (n=32)	Women (n=102)	*P* value
1. Because of your problem, do you feel depressed?			0.161
No	15 (46.9%)	29 (28.4%)	
Sometimes	7 (21.9%)	28 (27.5%)	
Yes	10 (31.2%)	45 (44.1%)	
2. Does stepping off the sidewalk worsen your problem?			**0.015**
No	21 (65.6%)	42 (41.2%)	
Sometimes	6 (18.8%)	18 (17.6%)	
Yes	5 (15.6%)	42 (41.2%)	
3. Because of your problem, is it difficult to concentrate?			**0.029**
No	23 (71.9%)	45 (44.1%)	
Sometimes	3 (9.4%)	23 (22.5%)	
Yes	6 (18.8%)	34 (33.3%)	
4. Because of your problem, is it difficult for you to walk around the house in the dark?			0.827
No	20 (62.5%)	56 (54.9%)	
Sometimes	4 (12.5%)	16 (15.7%)	
Yes	8 (25.0%)	30 (29.4%)	
5. Does turning over in bed worsen your problem?			0.137
No	7 (21.9%)	9 (8.8%)	
Sometimes	3 (9.4%)	15 (14.7%)	
Yes	22 (68.8%)	78 (76.5%)	
6. Because of your problem, do you restrict your travel for business or holidays?			0.154
No	27 (84.4%)	68 (66.7%)	
Sometimes	2 (6.2%)	19 (18.6%)	
Yes	3 (9.4%)	15 (14.7%)	
7. Does your problem affect your job or household responsibilities?			**0.001**
No	21 (65.6%)	33 (32.4%)	
Sometimes	7 (21.9%)	26 (25.5%)	
Yes	4 (12.5%)	43 (42.2%)	
8. Because of your problem, are you afraid to leave your home without having someone with you?			**0.024**
No	26 (81.2%)	59 (57.8%)	
Sometimes	4 (12.5%)	16 (15.7%)	
Yes	2 (6.2%)	27 (26.5%)	
9. Because of your problem, have you ever been embarrassed in front of others?			0.485
No	24 (75.0%)	72 (70.6%)	
Sometimes	7 (21.9%)	19 (18.6%)	
Yes	1 (3.1%)	11 (10.8%)	
10. Because of your problem, have you reduced your social activities such as going out to dinner, going to movies, or dancing at parties?			0.453
No	23 (71.9%)	60 (58.8%)	
Sometimes	3 (9.4%)	17 (16.7%)	
Yes	6 (18.8%)	25 (24.5%)	

Although we used the Spanish version of the questionnaire, we have included the original English questionnaire for comprehension purposes. Data shown as number and percentage of respondents

Statistically significant results are presented in bold

**Table 6:** Distribution of responses to the Dizziness Handicap Inventory – Screening version for primary care patients with symptoms of benign paroxysmal positional vertigo according to presence of nystagmus

	S-BPPV (n = 80)	O-BPPV (n = 54)	*P* value
1. Because of your problem, do you feel depressed?			0.485
No	23 (28.7%)	21 (38.9%)	
Sometimes	22 (27.5%)	13 (24.1%)	
Yes	35 (43.8%)	20 (37.0%)	
2. Does stepping off the sidewalk worsen your problem?			**0.038**
No	32 (40.0%)	31 (57.4%)	
Sometimes	13 (16.2%)	11 (20.4%)	
Yes	35 (43.8%)	12 (22.2%)	
3. Because of your problem, is it difficult to concentrate?			0.630
No	40 (50.0%)	28 (51.9%)	
Sometimes	14 (17.5%)	12 (22.2%)	
Yes	26 (32.5%)	14 (25.9%)	
4. Because of your problem, is it difficult for you to walk around the house in the dark?			0.278
No	42 (52.5%)	34 (63.0%)	
Sometimes	15 (18.8%)	5 (9.3%)	
Yes	23 (28.7%)	15 (27.8%)	
5. Does turning over in bed worsen your problem?			0.815
No	10 (12.5%)	6 (11.1%)	
Sometimes	12 (15.0%)	6 (11.1%)	
Yes	58 (72.5%)	42 (77.8%)	
6. Because of your problem, do you restrict your travel for business or holidays?			0.029
No	50 (62.5%)	45 (83.3%)	
Sometimes	17 (21.2%)	4 (7.4%)	
Yes	13 (16.2%)	5 (9.3%)	
7. Does your problem affect your job or household responsibilities?			0.070
No	31 (38.8%)	23 (42.6%)	
Sometimes	25 (31.2%)	8 (14.8%)	
Yes	24 (30.0%)	23 (42.6%)	
8. Because of your problem, are you afraid to leave your home without having someone with you?			0.668
No	48 (60.0%)	37 (68.5%)	
Sometimes	13 (16.2%)	7 (13.0%)	
Yes	19 (23.8%)	10 (18.5%)	
9. Because of your problem, have you ever been embarrassed in front of others?			0.461
No	56 (70.0%)	40 (74.1%)	
Sometimes	18 (22.5%)	8 (14.8%)	
Yes	6 (7.5%)	6 (11.1%)	
10. Because of your problem, have you reduced your social activities such as going out to dinner, going to movies, or dancing at parties?			**0.012**
No	41 (51.2%)	42 (77.8%)	
Sometimes	15 (18.8%)	5 (9.3%)	
Yes	24 (30.0%)	7 (13.0%)	

Although we used the Spanish version of the questionnaire, we have included the original English questionnaire for comprehension purposes. Data shown as number and percentage of respondents

Abbreviations: *O-BPPV* objective benign paroxysmal positional vertigo, *S-BPPV* subjective benign paroxysmal positional vertigo

Statistically significant results are presented in bold

**Table 7:** Distribution of responses to the Dizziness Handicap Inventory – Screening version for primary care patients with symptoms of benign paroxysmal positional vertigo according to a history of anxiety and/or depression

	No anxiety or depression (*n* = 83)	Anxiety and/or depression (*n* = 47)	*P* value
1. Because of your problem, do you feel depressed?			0.217
No	28 (33.7%)	15 (31.9%)	
Sometimes	26 (31.3%)	9 (19.1%)	
Yes	29 (34.9%)	23 (48.9%)	
2. Does stepping off the sidewalk worsen your problem?			0.660
No	36 (43.4%)	24 (51.1%)	
Sometimes	17 (20.5%)	7 (14.9%)	
Yes	30 (36.1%)	16 (34.0%)	
3. Because of your problem, is it difficult to concentrate?			0.444
No	46 (55.4%)	21 (44.7%)	
Sometimes	15 (18.1%)	9 (19.1%)	
Yes	22 (26.5%)	17 (36.2%)	
4. Because of your problem, is it difficult for you to walk around the house in the dark?			0.532
No	50 (60.2%)	24 (51.1%)	
Sometimes	12 (14.5%)	7 (14.9%)	
Yes	21 (25.3%)	16 (34.0%)	
5. Does turning over in bed worsen your problem?			0.295
No	8 (9.6%)	8 (17.0%)	
Sometimes	10 (12.0%)	8 (17.0%)	
Yes	65 (78.3%)	31 (66.0%)	
6. Because of your problem, do you restrict your travel for business or holidays?			1.000
No	59 (71.1%)	34 (72.3%)	
Sometimes	13 (15.7%)	7 (14.9%)	
Yes	11 (13.3%)	6 (12.8%)	
7. Does your problem affect your job or household responsibilities?			**0.042**
No	37 (44.6%)	15 (31.9%)	
Sometimes	24 (28.9%)	9 (19.1%)	
Yes	22 (26.5%)	23 (48.9%)	
8. Because of your problem, are you afraid to leave your home without having someone with you?			0.327
No	55 (66.3%)	26 (55.3%)	
Sometimes	13 (15.7%)	7 (14.9%)	
Yes	15 (18.1%)	14 (29.8%)	
9. Because of your problem, have you ever been embarrassed in front of others?			**0.035**
No	58 (69.9%)	36 (76.6%)	
Sometimes	20 (24.1%)	4 (8.5%)	
Yes	5 (6.0%)	7 (14.9%)	
10. Because of your problem, have you reduced your social activities such as going out to dinner, going to movies, or dancing at parties?			0.205
No	52 (62.7%)	29 (61.7%)	
Sometimes	9 (10.8%)	10 (21.3%)	
Yes	22 (26.5%)	8 (17.0%)	

Although we used the Spanish version of the questionnaire, we have included the original English questionnaire for comprehension purposes. Data shown as number and percentage of respondents

Statistically significant results are presented in bold

Patients with subjective BPPV (without nystagmus) reported greater perceived disability in stepping off the sidewalk (*p* = 0.038), traveling (p = 0.029), and participating in social activities (*p* = 0.012) (Table 6). No statistically significant differences were observed between patients with and without a history of anxiety and/or depression for responses to the question about feeling depressed (*p* = 0.217), although the former reported greater disability in relation to work and household responsibilities (*p* = 0.042) and feelings of embarrassment (*p* = 0.035) (Table 7).

## Discussion

This study shows that disability assessed by the DHI-S is significantly affected by posterior canal BPPV, particularly in women and in patients with subjective disease (vertigo but not nystagmus in the DH test).

The median age of the patients in this series, 52 years, is within the peak range for onset of BPPV (50–70 years) [1] and is also similar to ages described for patients in specialist settings [19, 23]. In primary care settings, median ages of 54.9 [24] and 61 years [25] have been reported.

There were over three times more women than men in our series (female to male ratio, 3.19). Women are often more numerous than men in studies of BPPV [2, 26–28], perhaps because the disease is more prevalent in women [2, 29, 30], including those in the 18–34 bracket [31].

Anxiety and depression were common and over 20% of the patients studied were being treated with benzodiazepines and/or antidepressants. These data are consistent with previous reports of high rates of affective disorders, such as anxiety, depression, demoralization, phobia, and somatization, in patients with BPPV [31, 32]. Kahraman et al. [33] recently showed that patients with BPPV may experience intense anxiety and/or panic disorder at the initial visit that may or may not improve with treatment. Attempts to explain why stressful events in the preceding year can trigger BPPV include the hypothesis that the increase in stress-related hormones triggered by the abnormal activation of the hypothalamus–pituitary–adrenal axis could interfere with inner ear blood flow and disrupt the calcium balance in the endolymph, critically affecting otoconial homeostasis [9, 34]. That said, higher depression scores in patients with BPPV have been attributed to the impact of symptoms on patients’ lives [10]. It is therefore perfectly plausible that anxiety and depression are both a cause and consequence of BPPV. In our sample, we found no differences in total scores on the DHI-S between patients with or without a history of anxiety and/or depression, even after adjusting for sex. In brief, thus, a previous diagnosis of anxiety and/or depression was not associated with worse disability results. We did, however, detect a considerable percentage of patients claiming that they felt depressed because of BPPV. Indeed, the fact that over 40% of patients reported that they sometimes felt depressed because of their vertigo adds to the doubts expressed in the recent update of the clinical BPPV guidelines about the adequacy of the term “benign” in benign paroxysmal positional vertigo in reference to the disease’s impact on quality of life, daily activities, and risk of falls [1].

Increased vertigo due to turning over in bed was by far the greatest problem reported by patients in the DHI-S. This is not surprising as this movement is known to trigger or increase episodes of vertigo and has a 90% specificity for the diagnosis of BPPV, although its sensitivity is low [35]. Onset on turning over in bed has also been identified as an independent predictor of BPPV diagnosed with the DH test (odds ratio, 4.36; 95% CI, 1.18–16.13) [36]. The second item with the highest number of “yes” responses was item 1 (“Because of your problem, do you feel depressed?). The median total score on the DHI-S, 16, was slightly lower than scores reported for specialist settings (mean, SD: 19.79 ± 10.,14) [19]; (17.19 ± 9.06) [37] although a similar score has also been reported (16,4 ± 10.71) [20]. It was also lower than the mean scores of 17.72 (SD = 9.98) and 22.67 (12.55) reported for patients with unilateral and bilateral Ménière disease, respectively [26, 38].

Women perceived greater disability as a result of BPPV than men. This finding is consistent with reports by Petri et al. [26] for patients with peripheral unilateral vestibular diseases, including BPPV. We were surprised to see that patients with subjective posterior canal BPPV reported greater levels of perceived disability. This observation, however, should be interpreted with caution because, as mentioned in the introduction, significant differences between patients with subjective and objective BPPV have not been found for demographics, clinical characteristics, or status before and after repositioning procedures [7, 39]. Just one study in Argentina reported higher scores (greater disability) in the physical subscale of the original DHI for patients with nystagmus and in the emotional subscale for patients without nystagmus. There were no significant differences, however, in total scores [40].

The higher proportion of women in our study could be the result of inclusion bias, but it could also reflect the fact that posterior canal BPPV is more common in women and that in Spain, adult women are more likely to visit primary care facilities than men [41].

We acknowledge some limitations of this study. The proportion of patients with subjective BPPV in our series was also higher than that reported in the literature, possibly because neither Frenzel goggles nor videonystagmography were used to detect nystagmus in the DH test to create a more realistic primary care diagnostic environment. However, in mild cases, nystagmus can be difficult to observe, especially given the vertical nystagmus component suppression that can occur in room light. Therefore, the study’s results of greater perceived disability in patients with Subjective-BPPV can be challenged. Given the complexity of interpreting nystagmus during the Dix-Hallpike test, the training received may have been too short. A longer workshop could improve the diagnostic and therapeutic accuracy of GPs, especially to detect and interpret nystagmus correctly in DHT.

Results must be interpreted in light of the descriptive nature of the paper. Significant tests were performed independently with no adjustment for multiple hypotheses testing. In fact, given the large amount of contrasts, significance corrections would have led to only two tests finding statistically significant differences: DHI Total Score and the 7th Item by sex. Studies designed to specifically test each of the “independently significant” contrast would be required to confirm statistical significance.

## Conclusions

BPPV had a negative impact on the quality of life of patients, particularly women and patients with subjective disease (vertigo without nystagmus). The greatest perceived difficulties were related to feelings of depression and turning over in bed. The results of this study help to confirm the importance of early diagnosis and effective treatment in patients with BPPV. Both diagnosis and treatment are possible in primary care. More research is needed on how BPPV affects the quality of life of patients attending primary care and on how repositioning maneuvers can help to alleviate these effects. It would also be interesting to explore differences between subjective and objective BPPV, particularly in terms of perceived disability, and to conduct more studies using the shorter, simpler DHI-S scale.

## Data Availability

All data generated or analyzed during this study are included in this published article [and its supplementary information files].

## Abbreviations

BPPV: Benign paroxysmal positional vertigo
DH test: Dix Hallpike test
DHI: Dizziness Handicap inventory
DHI-S: Dizziness Handicap inventory screening version
DHI-SF: Dizziness Handicap inventory short form
GPs: General practitioners
HRQOL: Health-related quality of life
ICS: Catalan Institute of Health
IDIAP: Institute for Research in Primary Care
IQR: Interquartile Range
O-BPPV: Objective benign paroxysmal positional vertigo
PC: Primary Care
S-BPPV: Subjective benign paroxysmal positional vertigo
USR: Research Support Unit
